# Pectate Lyase from Fusarium sacchari Induces Plant Immune Responses and Contributes to Virulence

**DOI:** 10.1128/spectrum.00165-23

**Published:** 2023-05-04

**Authors:** Caixia Wang, Zhen Huang, Zhenzhen Duan, Lixiang Zhu, Ruolin Di, Yixue Bao, Charles A. Powell, Qin Hu, Baoshan Chen, Muqing Zhang, Wei Yao

**Affiliations:** a State Key Lab for Conservation and Utilization of Subtropical Agri-Biological Resources, Guangxi Key Lab of Sugarcane Biology, Guangxi University, Nanning, China; b IRREC-IFAS, University of Florida, Fort Pierce, Florida, USA; USDA - San Joaquin Valley Agricultural Sciences Center

**Keywords:** Pokkah Boeng disease, *Fusarium sacchari*, pectate lyase, plant immunity, pathogenicity

## Abstract

Fusarium sacchari is one of the primary pathogens causing Pokkah Boeng disease (PBD) in sugarcane in China. Pectate lyases (PL), which play a critical role in pectin degradation and fungal virulence, have been extensively studied in major bacterial and fungal pathogens of a wide range of plant species. However, only a few PLs have been functionally investigated. In this study, we analyzed the function of the pectate lyase gene, *FsPL*, from F. sacchari*. FsPL* is a key virulence factor of F. sacchari and can induce plant cell death. *FsPL* also triggers the pathogen-associated molecular pattern (PAMP)-triggered immunity (PTI) response in Nicotiana benthamiana, as reflected by increases in reactive oxygen species (ROS) production, electrolyte leakage, and callose accumulation, as well as the upregulation of defense response genes. In addition, our study also found that the signal peptide of *FsPL* was necessary for induced cell death and PTI responses. Virus-induced gene silencing showed that *FsPL*-induced cell death in Nicotiana benthamiana was mediated by leucine-rich repeat (LRR) receptor-like kinases BAK1 and SOBIR1. Thus, *FsPL* may not only be a critical virulence factor for F. sacchari but may also induce plant defense responses. These findings provide new insights into the functions of pectate lyase in host-pathogen interactions.

**IMPORTANCE** Pokkah Boeng disease (PBD) is one of the main diseases affecting sugarcane in China, seriously damaging sugarcane production and economic development. Therefore, it is important to clarify the pathogenic mechanisms of this disease and to provide a theoretical basis for the breeding of PBD-resistant sugarcane strains. The present study aimed to analyze the function of *FsPL*, a recently identified pectate lyase gene from F. sacchari. *FsPL* is a key virulence factor of F. sacchari that induces plant cell death. Our results provide new insights into the function of pectate lyase in host-pathogen interactions.

## INTRODUCTION

Pokkah Boeng disease (PBD), a fungal disease of sugarcane caused by Fusarium sacchari and other Fusarium species, mainly attacks young leaves and stem tips (1, 2). PBD-affected sugarcane leaves exhibit chlorosis and distortion during the early stages of infection, and the infection may progress to necrosis and top rot, even killing the plant itself (1). PBD is one of the main diseases affecting sugarcane in China, seriously damaging sugarcane production and economic development (1). However, despite the importance of this disease, few previous studies have investigated the pathogenic mechanisms underlying F. sacchari invasion. It is therefore important to clarify these pathogenic mechanisms to provide a theoretical basis for the breeding of PBD-resistant sugarcane strains.

The plant cell wall, a complex network of polysaccharides, including cellulose, hemicellulose, and pectin (3–5), is the initial physical and defensive barrier against a variety of biological stresses and is thus an important site of host-pathogen interaction (5, 6). Plant cell walls usually participate in the immune response via dynamic remodeling to prevent pathogen infection (7); the plant cell wall also senses external stressors and transmits signals to stimulate defensive responses in the host (7–9). To successfully infect a host, plant pathogens produce a series of cell wall-degrading enzymes (CWDEs), including cellulase, pectinase, xylanase, and xyloglucanase, which disintegrate the cell wall, allowing pathogens to penetrate host cells and spread throughout the plant (5). Plant defense responses include pathogen-associated molecular pattern (PAMP)-triggered immunity (PTI) and effector-triggered immunity (ETI) (10). In PTI, pattern recognition receptors (PRRs) on the surfaces of plant cells recognize PAMPs or damage-associated molecular patterns (DAMPs), triggering PTI reactions (9). Typical PTI reactions include bursts of reactive oxygen species (ROS), callose accumulation, and electrolyte leakage, which typically confine microorganisms to the site of infection (9). Oligogalacturonic acids, which are produced by the pectate lyase cleavage of polygalacturonic acid via a β-elimination reaction, are recognized by the plasma membrane-associated protein WAK1 on the plant cell surface, activating the MAPK cascade; the MAPK cascade increases free cytosolic calcium levels and promotes ROS generation, triggering PTI responses (11–14).

However, pathogens can interfere with or inhibit PTI by secreting effector proteins that enhance pathogen virulence. In response, plants recruit R proteins that recognize these effectors directly or indirectly, triggering ETI, the second-line defense response (10). One of the most important roles of plant ETI is to trigger the hypersensitive response (HR), which produces defense proteins that induce rapid programmed cell death (PCD) at the site of pathogen attack to limit pathogen transmission. This division between PAMPs and effectors, or between PTI and ETI, is, however, blurred and has been challenged recently (15).

The brassinosteroid insensitive 1 (BRI1)-associated receptor kinase 1 (BAK1) and the LRR receptor-like kinase (LRR-RLK)-like suppressor of BIR1-1 (SOBIR1) are important regulatory components that are required for functional immune signaling on the plant plasma membrane. These molecules can identify some extracellular PAMPs and apoplastic pathogen effectors through membrane-bound receptor-like kinases (RLKs) and/or receptor-like proteins (RLPs), thus participating in multiple PRR and signaling activation pathways (16–19).

Pectate lyase is an important enzyme secreted by plant pathogens that has been reported in a variety of microorganisms, including bacteria (e.g., Erwinia carotovora, Erwinia chrysanthemi, and Pseudomonas solanacearum) (20–22) and fungi (e.g., Verticillium dahlia, Valsa mali, and Colletotrichum coccodes) (23–25). Previously, we cloned the pectate lyase gene of F. sacchari (*FsPL*, Fs04471) (see Fig. S1 in the supplemental material) and found that this gene was upregulated during the interaction between F. sacchari and sugarcane (23). Further analysis showed that *FsPL* encoded a typical fungal effector protein with a secretory signal peptide (23).

However, the specific functions of the *FsPL* gene in F. sacchari and in plant hosts remain unknown. Therefore, in this paper, we aimed to further explore the function of the *FsPL* gene. Our results showed that *FsPL* is an important virulence factor of F. sacchari and that the knockout of *FsPL* significantly decreases virulence. Moreover, FsPL requires the signal peptide to induce SOBIR1-BAK1-dependent cell death in N. benthamiana. We also found that *FsPL* triggers the typical PTI response and that *FsPL* induced signal peptide-dependent cell necrosis in maize leaves. Our findings will provide new insights into the functions of pectate lyase in host-pathogen interactions.

## RESULTS

### Construction of the *FsPL* deletion strains.

To investigate the roles of the *FsPL* gene in F. sacchari, we generated *FsPL* deletion mutants (ΔFsPL), in which the entire open reading frame (ORF) of *FsPL* was replaced with a hygromycin gene by homologous recombination (Fig. S2a). PCR amplifications of *FsPL* in the wild type (WT) and the transformants using specific primers confirmed that the *FsPL* gene had been deleted in the transformants and replaced with the *hyg* gene (Fig. S2b). The long recombinational sequence (A-*HYG*-B) from the transformants was cleaved and verified using MluI (Fig. S2c). When hybridized with probes derived from the ORF of the *FsPL* gene (probe 1), the fragment corresponding to the *FsPL* gene was present in the wild type but absent in the deletion mutants. In addition, a band of the expected size was present in the deletion mutants when hybridized with the *Hyg* probe (probe 2), indicating that the deletion mutants had a single-locus homologous recombination at the location of the *FsPL* gene (Fig. S2d).

### FsPL is an important virulence factor for F. sacchari.

**FIG 1:**
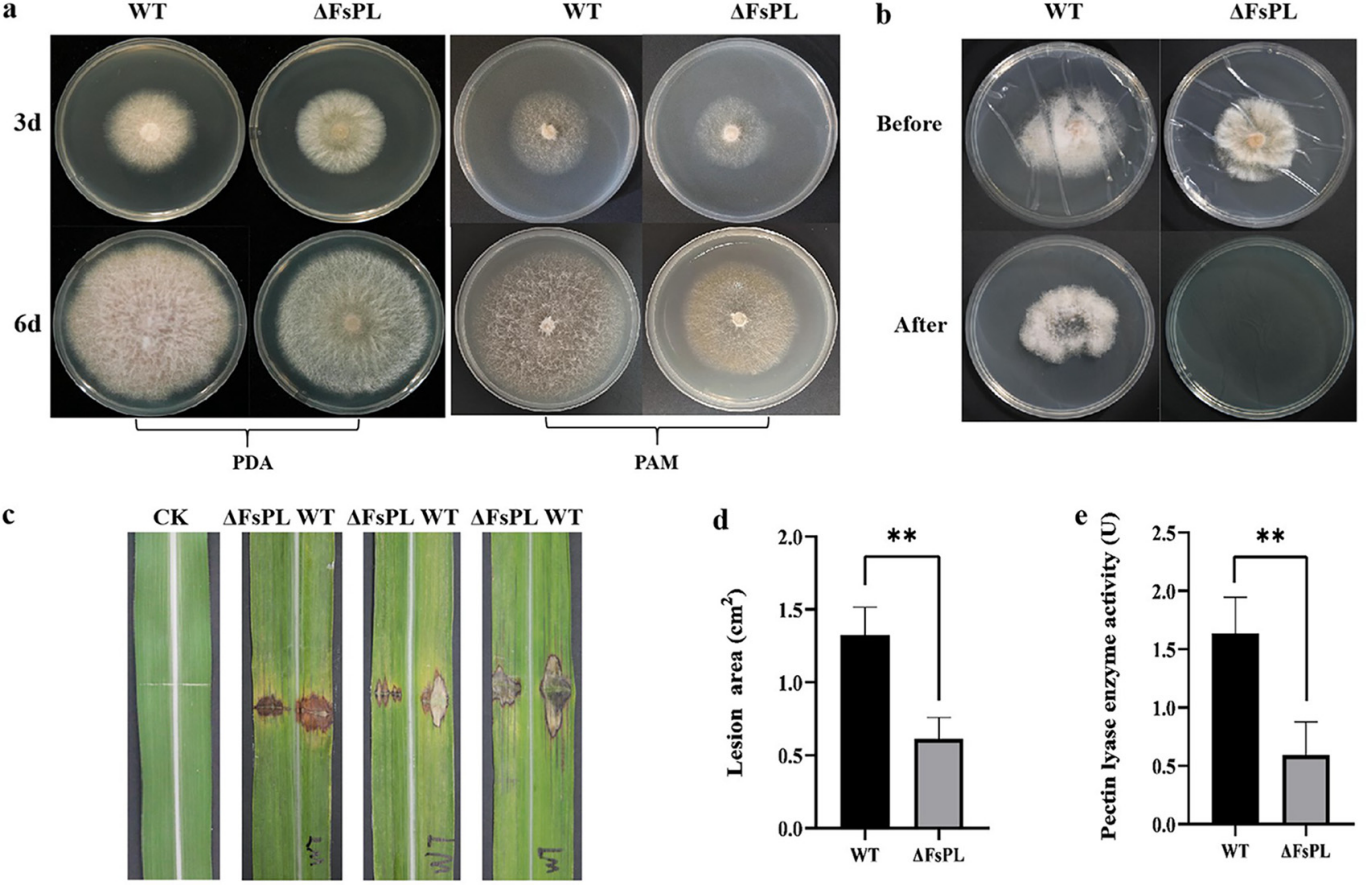
Biological phenotype and pathogenicity of the Fusarium sacchari *FsPL* deletion mutant (ΔFsPL). (a) Growth of ΔFsPL and wild-type F. sacchari (WT) in potato dextrose agar (PDA) medium and pectin agar medium (PAM). Photographs were taken after 3 days and 6 days of culture. (b) Puncture ability of ΔFsPL and wild-type F. sacchari, as demonstrated by the cellophane penetration test. “Before” corresponds to 3 days of growth on cellophane, and “after” corresponds to 3 days of growth after the cellophane was removed. (c) Leaves of sugarcane cultivar Zhongzhe 1 15 days after surface scratches were inoculated with ΔFsPL and WT. The area of each lesion was determined from the images using ImageJ software (50). (d) Average lesion area per sugarcane leaf 15 days after inoculation with ΔFsPL and WT. (e) Average pectate lyase activity in ΔFsPL and WT, as determined using DNS colorimetry. In panels d and e, error bars indicate standard error (*n* = 3; **, *P* < 0.01).

In potato dextrose agar (PDA) medium, the F. sacchari *FsPL* deletion mutant (ΔFsPL) had a slightly lower hyphal growth rate (Fig. S3a) and produced significantly fewer aerial hyphae than wild-type F. sacchari (Fig. 1a). In pectin agar medium (PAM), in which pectin was the only carbon source, the mutant colony was significantly smaller than that of the wild type (Fig. 1a and Fig. S3b). This phenomenon suggested that the *FsPL* gene might be involved in the degradation of host pectin by pathogenic fungi. However, spore morphology and sporulation quantity did not differ significantly between the wild type and the mutant (Fig. S3c and d). *In vivo* tests of pathogenicity using the sugarcane cultivar Zhongzhe 1 showed that pathogenicity of the deletion mutant was reduced compared to the wild type: the necrotic lesions on the sugarcane leaves inoculated with the ΔFsPL mutant were significantly smaller than those inoculated with wild-type F. sacchari (Fig. 1c and d); similar observations were made after *in vitro* inoculation (Fig. S3e and f). Because the pathogenicity of plant-pathogenic fungi is generally correlated with the ability to penetrate the host epidermis (24), we performed cellophane penetration tests to determine whether the loss of the *FsPL* gene affected the invasive ability of the F. sacchari mycelia. As expected, the *ΔFsPL* deletion mutants, unlike wild-type F. sacchari, were unable to penetrate cellophane (Fig. 1b). Therefore, we speculated that the *FsPL* gene affected F. sacchari’s pathogenicity by improving the penetration ability of the F. sacchari mycelia. Subsequently, the extracellular pectate lyase activity levels of the wild type and the mutant were determined using the dinitrosalicylic acid (DNS) method. We found that the extracellular pectate lyase activity of the mutant was significantly lower than that of wild-type F. sacchari (Fig. 1e). These results indicated that the deletion of the *FsPL* gene affected the extracellular pectate lyase activity of F. sacchari.

### Transient expression of *FsPL* in N. benthamiana induces cell death.

**FIG 2:**
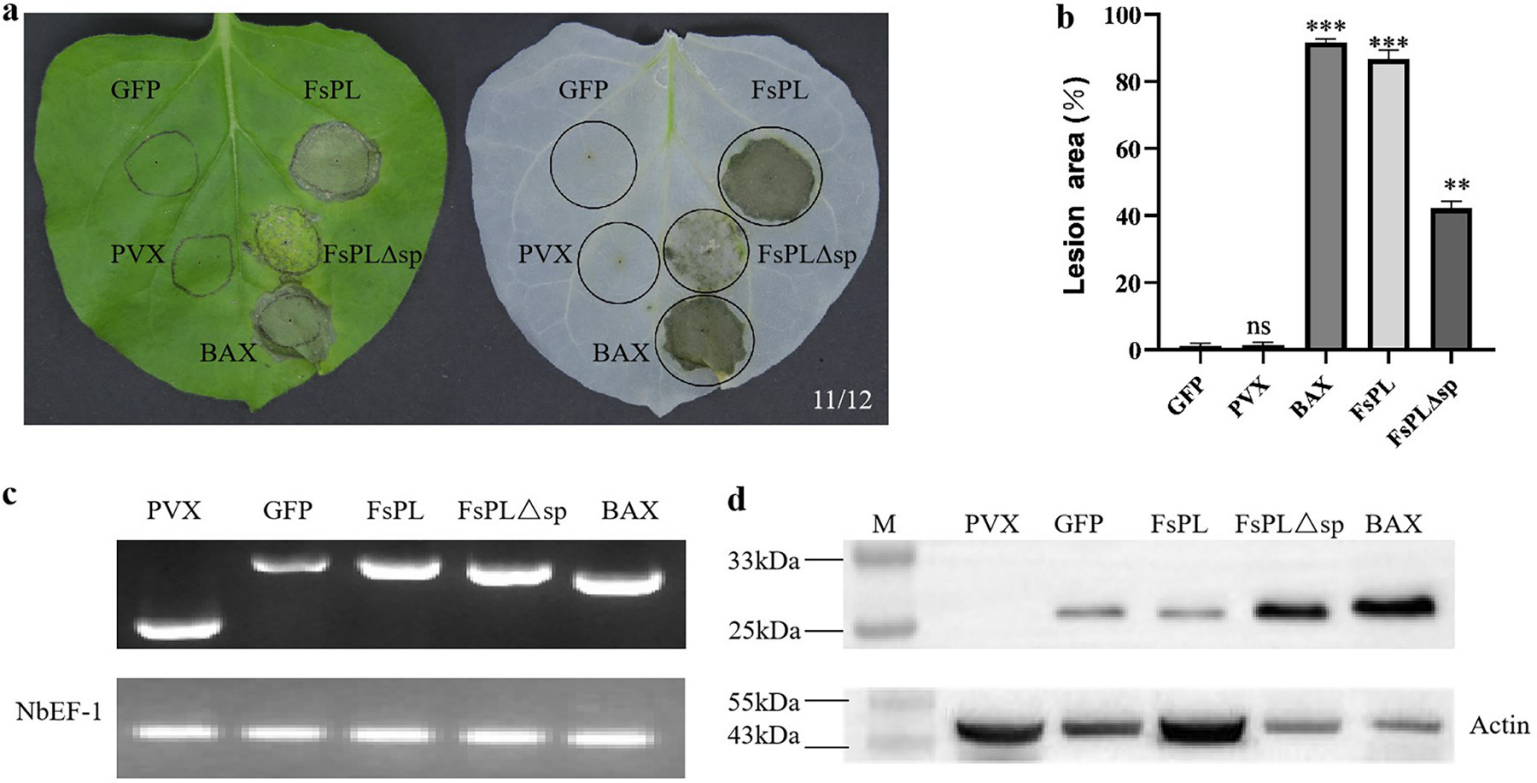
Transient expression of *FsPL* induces a PCD response in N. benthamiana leaves. (a) Representative images of N. benthamiana leaves inoculated with full-length *FsPL* (*FsPL*) and *FsPL* lacking the signal peptide (*FsPL*Δsp) before (left) and after (right) ethanol decolorization. Leaves were inoculated with the empty vector (PVX) and the recombinant empty vector (GFP) as negative controls and with Bcl-2-associated X protein (BAX), which induces cell necrosis in N. benthamiana leaves, as a positive control. Picture digital representation shows the number of times a similar result was detected from total repeats. (b) Average lesion area corresponding to each inoculate. Values shown are the means ± standard error of three independent experiments (ns, *P* > 0.05; **, *P* < 0.01; ***, *P* < 0.001). (c) Representative electrophoresis gel demonstrating the successful transcription of the target genes at the inoculation sites in the N. benthamiana leaves. *NbEF-1* was used as the internal reference. (d) Representative Western blotting demonstrating the successful expression of the corresponding target proteins at the inoculation sites in the N. benthamiana leaves. Actin was used as the internal reference.

Like the positive control (PVX-BAX), *Agrobacterium-*mediated transient expression of *FsPL* (PVX-FsPL) induced cell necrosis in N. benthamiana leaves (Fig. 2a). However, the necrotic effects of *FsPL* lacking the signal peptide (PVX-FsPLΔsp) were less marked (Fig. 2a). No lesions were observed after inoculation with the negative-control vectors (PVX-EV and PVX-GFP) (Fig. 2a). Indeed, quantitative comparisons of the lesion area showed that the expression of *FsPL* led to a similar degree of necrosis in N. benthamiana leaves as the expression of Bcl-2-associated X protein (BAX), while the degree of necrosis associated with the expression of *FsPL* lacking the signal peptide was significantly reduced (Fig. 2b). Real-time PCRs (RT-PCRs) confirmed that the target genes were successfully transcribed in N. benthamiana leaves (Fig. 2c), while Western blot analysis confirmed that the corresponding proteins were successfully expressed (Fig. 2d). These results suggested that the expression of *FsPL* initiated a PCD in the host and that the presence of the signal peptide was required for full function.

### *FsPL* triggers PTI responses in N. benthamiana leaves.

**FIG 3:**
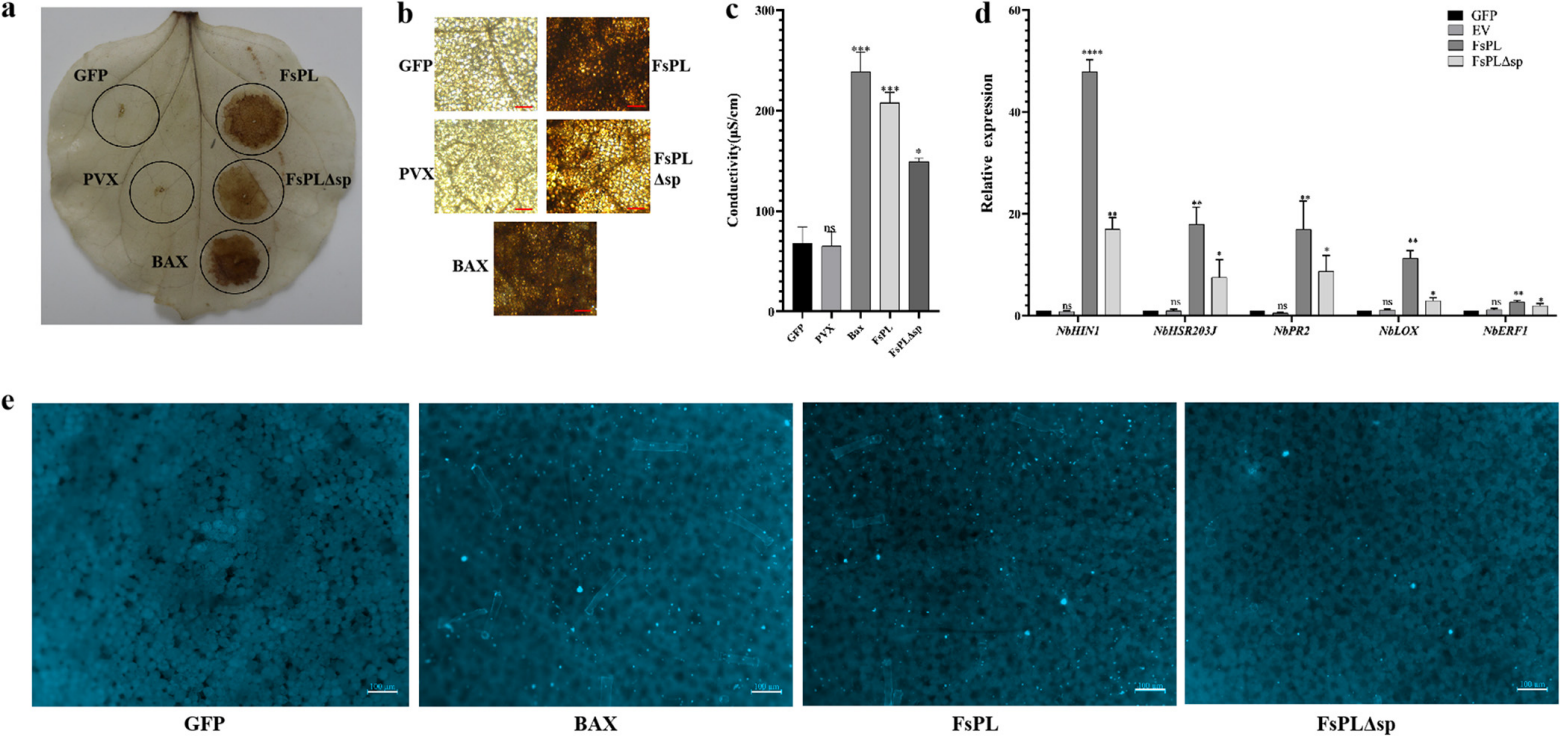
*FsPL* expression induces PTI responses in N. benthamiana leaves. (a) Representative image of an N. benthamiana leaf after inoculation with full-length *FsPL* (*FsPL*) and *FsPL* lacking the signal peptide (*FsPL*Δsp), DAB staining, and ethanol decolorization, which color intensity reflects ROS accumulation. Leaves were inoculated with the empty vector (PVX) and the recombinant empty vector (GFP) as negative controls and with Bcl-2-associated X protein (BAX), which induces cell necrosis in N. benthamiana leaves, as a positive control. (b) Magnification of the lesions shown in panel a. Scale bars, 500 μm. (c) Electrical conductivity at the inoculation sites in the N. benthamiana leaves 48 h after infiltration. Error bars indicate the standard error of three independent experiments (ns, *P* > 0.05; *, *P* < 0.05; ***, *P* < 0.001). (d) Expression patterns of marker genes of the hypersensitive response (*NbHIN1* and *NbHSR203J*) and immune defense (*NbLOX*, *NbERF1*, and *NbPR2*) at *FsPL-* and *FsPLΔ*sp-infiltrated sites in N. benthamiana 48 h after infiltration. GFP and the empty vector were used as negative controls. *NbEF-1* was used as the internal reference gene. Error bars indicate the standard error of three independent experiments (ns, *P* > 0.05; *, *P* < 0.05; ***, *P* < 0.001). (e) Representative images showing aniline blue staining, which reflects callose deposition, at the GFP-, BAX-, *FsPL-*, and *FsPLΔsp-*infiltrated sites in N. benthamiana leaves 48 h after infiltration. Scale bars, 100 μm.

Three markers of the PTI response to pathogen invasion in plants are ROS production, electrolyte leakage, and callose accumulation. We used 3′3-diaminobenzidine (DAB) staining to detect ROS accumulation at the inoculation sites in the N. benthamiana leaves. An equivalent degree of brown staining, indicating similar levels to ROS accumulation, was observed at the BAX and *FsPL* inoculation sites, indicating that, like BAX, *FsPL* caused ROS eruption in the leaf tissues of N. benthamiana (Fig. 3a and b). The intensity of ROS staining was decreased at the sites inoculated with *FsPLΔ*sp (Fig. 3a and b).

Levels of electrical conductivity, which reflect electrolyte leakage, were significantly greater at the leaf sites inoculated with *FsPL*, BAX, and *FsPLΔsp* than at the sites inoculated with the control vectors (PAX and PAX-GFP) (Fig. 3c). Notably, although there was no significant difference in conductivity between the BAX- and *FsPL-*inoculated sites, conductivity levels at the sites inoculated with *FsPLΔ*sp were lower (Fig. 3c).

Aniline blue staining showed that callose accumulation was greater at the BAX-, *FsPL-*, and *FsPLΔsp-*inoculated sites than at the green fluorescent protein (GFP)-inoculated site and that callose accumulation was decreased at the *FsPLΔ*sp-inoculated site compared to the BAX- and *FsPL-*inoculated sites (Fig. 3e). These results suggested that *FsPL* expression induced typical PTI responses in the leaves of N. benthamiana and that the removal of the signal peptide reduced this response.

### *FsPL* triggers the upregulation of genes associated with the immune response in N. benthamiana leaves.

The upregulation of immune-related genes is an important indicator of the initiation of the plant immune response (13, 25). The genes *NbHIN1* and *NbHSR203J*, which are markers of the plant hypersensitive immune response (13), were dramatically upregulated after *FsPL* infiltration (*P* < 0.001) (Fig. 3d). *NbLOX*, *NbPR2*, and *NbERF1*, which are used as markers of jasmonic acid (JA), salicylic acid (SA), and ethylene-dependent immunity, respectively (26), were also significantly upregulated after *FsPL* infiltration (*P* < 0.01) (Fig. 3d). Although *NbHIN1*, *NbHSR203J*, *NbLOX*, *NbPR2*, and *NbERF1* were significantly upregulated after *FsPLΔsp* infiltration compared to the controls, the degree of upregulation was significantly lower than that after *FsPL* infiltration (*P* < 0.05) (Fig. 3d). These results suggested that *FsPL* may influence the immune response induced by plant hormones and that the strength of this influence is decreased when the signal peptide is lost.

### *SOBIR1* and *BAK1* are required for *FsPL*-induced cell death in N. benthamiana leaves.

**FIG 4:**
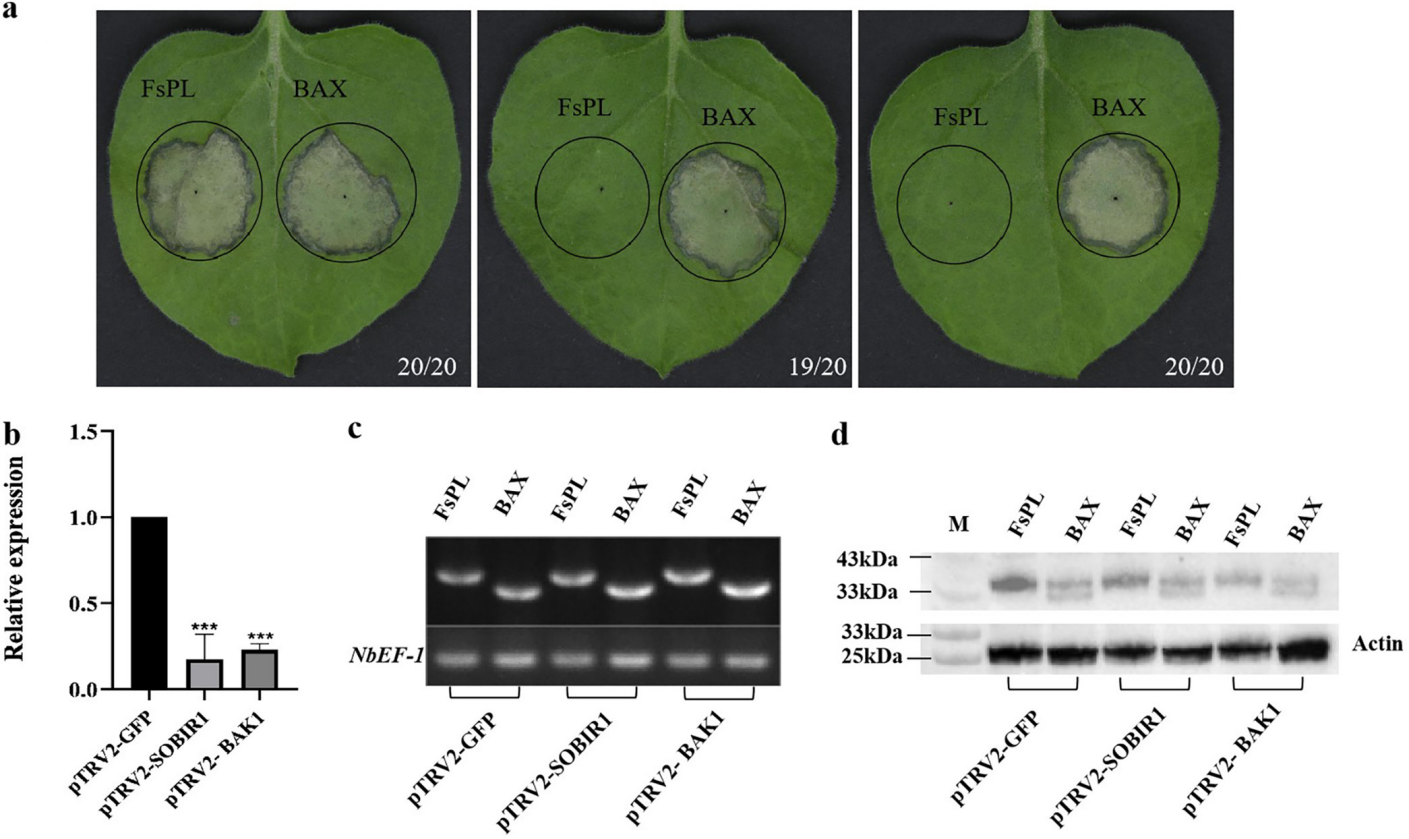
BAK1 and SOBIR1 mediate *FsPL*-triggered cell death in N. benthamiana. (a) Representative images showing N. benthamiana leaves from control (pTRV2-GFP), *SORBIR1*-silenced (pTRV2-SORBIR1), and *BAK1*-silenced (pTRV-BAK1) plants. Leaves were photographed 7 days after infiltration with *FsPL* or BAX (the positive control). Picture digital representation shows the number of times a similar result was detected from total repeats. (b) The efficiency of VIGS-driven BAK1 and SOBIR1 silencing, based on qRT-PCR analysis. Values represent the means ± standard deviations of three independent replicates (***, *P* < 0.001). (c) Representative electrophoresis gel demonstrating the successful transcription of the target genes *FsPL* and BAX at the inoculation sites in the leaves of control (pTRV2-GFP), *SORBIR1*-silenced (pTRV2-SORBIR1), and *BAK1*-silenced (pTRV-BAK1) N. benthamiana. *NbEF-1* was used as the internal reference. (d) Western blotting demonstrating the successful expression of the corresponding target proteins at the inoculation sites in the N. benthamiana leaves. Actin was used as the internal reference.

To determine whether the immune pathway genes *SOBIR1* and *BAK1* participate in *FsPL*-induced cell death, we silenced the *NbSOBIR1* or *NbBAK1* gene in N. benthamiana using VIGS. Quantitative RT-PCR (qRT-PCR) analysis confirmed that *BAK1* and *SOBIR1* were significantly downregulated in the *BAK1*- and *SOBIR1*-silenced N. benthamiana lines, respectively (Fig. 4b). We found that *FsPL* failed to induce cell death in both *BAK1*- and *SOBIR1*-silenced N. benthamiana, while BAX infiltration continued to induce cell death in the gene-silenced lines (Fig. 4a). RT-PCRs confirmed that the target genes *FsPL* and *BAX* were successfully transcribed in N. benthamiana leaves (Fig. 4c), while Western blot analysis confirmed that the corresponding proteins were successfully expressed (Fig. 4d). Together, these results indicated that *SOBIR1* and *BAK1* mediate *FsPL*-triggered cell death in N. benthamiana.

### Overexpression of *FsPL* in maize leads to PCD response.

**FIG 5:**
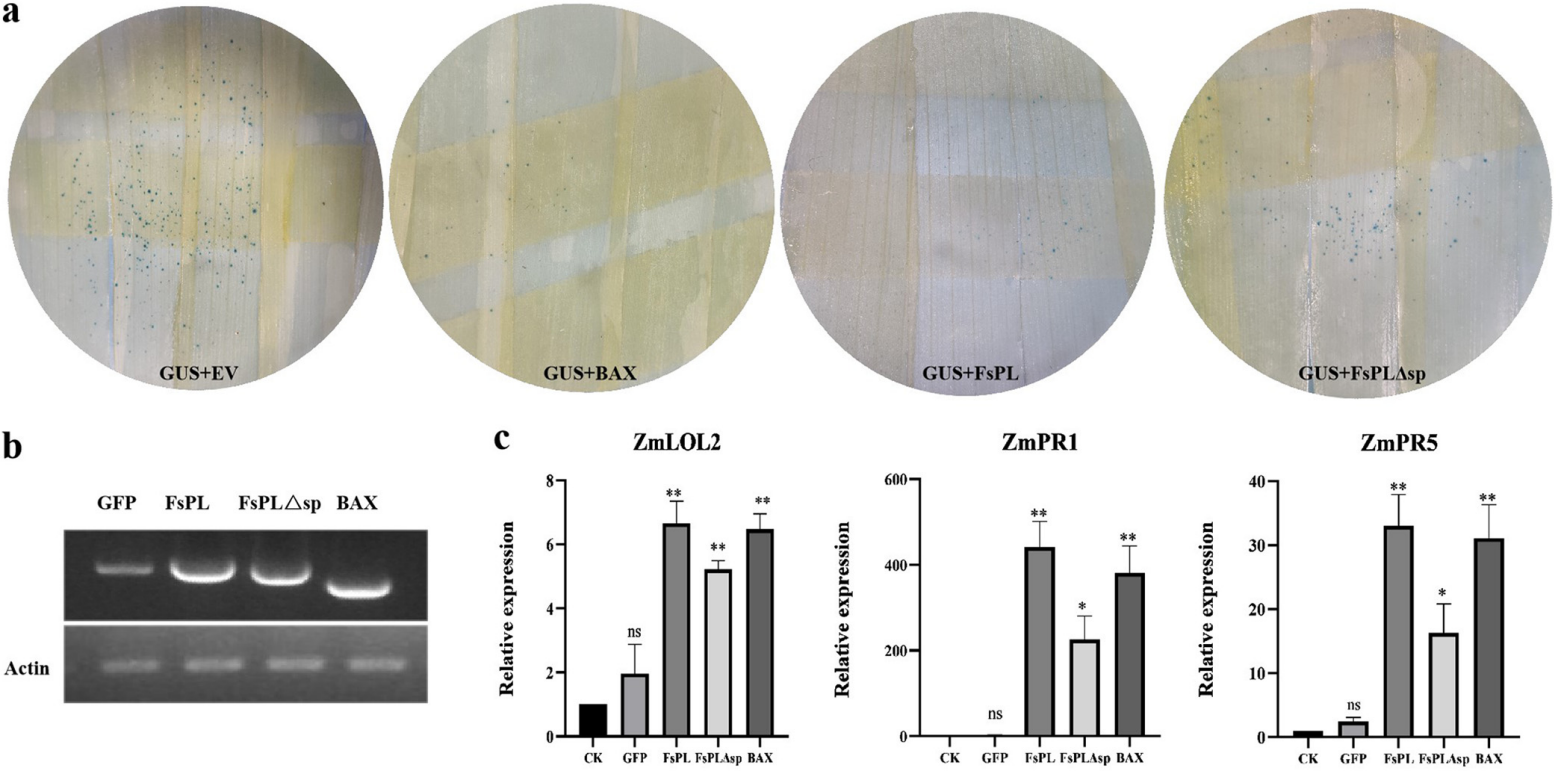
Overexpression of *FsPL* in maize leaves using particle bombardment triggers the PCD response. (a) Maize leaves were bombarded with the indicated mixtures of DNA. We used the pCAMBIA2300-GUS plasmid (2 μg) plus the empty vector (2 μg) as the negative control (GUS+EV), the pCAMBIA2300-GUS plasmid (2 μg) plus the BAX plasmid (2 μg) as the positive control (GUS+BAX), and the GUS plasmid (2 μg) plus the FsPL plasmid (2 μg) or the FsPLΔsp plasmid (2 μg) as the experimental group (GUS+FsPL/FsPLΔsp). The number of blue dots reflects the number of living cells, and decreases in the number of blue dots compared with the control (GUS+EV) indicate that the target gene causes cell death. (b) Blots showing the RT-PCR results; actin was used as the internal reference. (c) Expression patterns of the marker genes for PCD (*ZmLOL2*) and SAR (*ZmPR1* and *ZmPR5*) at *FsPL-* and *FsPLΔ*sp bombardment sites in maize 48 h after bombardment. Unbombarded blades were used as the blank control (CK), GFP was used as the negative control, and BAX was used as the positive control. Actin was used as the internal reference gene. Error bars represent the standard error of three independent experiments (ns, *P* > 0.05; *, *P* < 0.05; **, *P* < 0.01).

To further verify the function of the *FsPL* gene, we overexpressed *FsPL* in maize, a close relative of sugarcane (the natural host of F. sacchari), using a single-barreled particle bombardment with *GUS* as a reporter gene. After particle bombardment, many blue spots (corresponding to living cells) appeared on the negative-control leaves bombarded with the empty vector, indicating that particle bombardment was successful (Fig. 5a). The number of blue spots decreased by approximately 89% after bombardment with the BAX vector, demonstrating that BAX, the positive control, induced PCD in the maize leaves (Fig. 5a; Table 1). In comparison to the negative control, the number of blue spots on leaves bombarded with *FsPL* and *FsPLΔsp* decreased by about 76% and about 52%, respectively (Fig. 5a; Table 1), indicating that *FsPL* induced a PCD response similar to that induced by BAX and that the PCD response induced by *FsPL* without the signal peptide was weakened.

**TABLE 1:** Induction of programmed cell death in maize via the transient expression of *FsPL* using particle bombardment

Mixture in barrel 1^*a*^	Mixture in barrel 2^*a*^	Log ratio^*b*^	*P* value^*c*^
GUS + BAX	GUS + EV	0.11 ± 0.02	<0.01
GUS + FsPL	GUS + EV	0.24 ± 0.04	<0.01
GUS + FsPLΔsp	GUS + EV	0.48 ± 0.04	<0.05

a Barrels 1 and 2 were physically identical, and the masses of DNA in each barrel were identical. Three biological replicates were performed per combination in maize leaves with the same growth pattern.

b Log ratios of the blue spots in barrel 1 compared to barrel 2; data shown are the means ± standard error of three biological replicates.

c *P* values were calculated from the log ratios using two-way ANOVAs.

The gene *ZmLOL2*, which is a marker of the plant PCD response, was dramatically upregulated after *FsPL* expression (Fig. 5c). *ZmPR1* and *ZmPR5*, which are markers of plant systemic-acquired resistance (SAR), were also significantly upregulated after *FsPL* expression (Fig. 5c). Although *ZmLOL2*, *ZmPR1*, and *ZmPR5* were significantly upregulated after *FsPL*Δsp expression compared to the control, the degree of upregulation was lower than that after *FsPL* expression (Fig. 5c). RT-PCRs confirmed that the target genes were successfully transcribed in maize leaves (Fig. 5b). These results suggested that *FsPL* may induce PCD and SAR in maize and that the strength of this inducement is decreased when the signal peptide is lost.

## DISCUSSION

Plant cell walls are the first barrier to pests and diseases. To overcome this barrier, pathogenic fungi secrete a number of CWDEs that destroy the structure of the plant cell wall, allowing infection to progress (27). Specifically, CWDEs hydrolyze the main components of the plant cell wall, depolymerizing and softening the cells and relaxing intercellular spaces, thus facilitating invasion and nutrient uptake by pathogenic microorganisms (5). Recent studies have revealed that several CWDEs functioned as virulence factors in plant pathogens and were also recognized as PAMPs by plant PRRs to trigger the PTI responses during plant-pathogen interactions (28). In this study, we analyzed the function of the F. sacchari pectate lyase gene *FsPL*. We found that *FsPL* functions as a virulence factor that contributes to F. sacchari virulence in host plants. *FsPL* had the ability to induce cell death and plant PTI responses dependent on its signal peptides. Furthermore, the *FsPL* death-inducing signal was mediated by the plant LRR receptor-like kinases (RLKs) BAK1 and SOBIR1. Our data suggested that *FsPL* contributes to F. sacchari virulence and induced plant defense responses.

Gene knockout experiments have proved that CWDEs secreted by a large number of pathogenic fungi are involved in the pathogenic process. For instance, disruption of the pectate lyase gene *pelB* in Colletotrichum gloeosporioides reduced the virulence of the mutant by about 40% (29), while knockdown of the pectate lyase gene *PL1332* in Alternaria brassicicola reduced the virulence of the mutant by about 30% (30). Similarly, knockdown of two *PL* genes in Nectria hematococca resulted in a complete loss of pathogenicity in the double-knockout mutants (31).

To investigate the functions of the F. sacchari *PL* gene (*FsPL*), we constructed *FsPL* knockout F. sacchari mutants. The *FsPL* knockout mutant had a marked decline in hyphal growth rate in pectin agar medium and produced significantly fewer aerial hyphae than wild-type F. sacchari, but the spore morphology and sporulation quantity were similar between the mutant and the wild type. This indicated that *FsPL* may participate in the vegetative growth of pathogenic fungi, as well as in the degradation of host pectin by pathogen fungi, but not in the regulation of sporulation. Similarly, deletion of the pectate lyase gene *MoPL1* affected the growth of Magnaporthe oryzae (32), while deletion of the pectate lyase gene *VdPL1-4* affected neither the spore morphology nor the spore production of Verticillium dahliae (33). *In vitro* and *in vivo* inoculation experiments showed that the virulence of the *FsPL* knockout mutant was significantly reduced compared to wild-type F. sacchari. It has been shown that the deletion of CWDE genes reduces the penetration ability, and thus the pathogenicity, of pathogenic fungi. For example, disruption of cutinase gene expression reduced the penetrative ability of Fusarium solani (34). Similarly, the *FsPL* knockout mutant, unlike wild-type F. sacchari, could not penetrate cellophane, suggesting that the deletion of the *FsPL* gene decreased the penetration ability of the F. sacchari mycelia, thus reducing the pathogenicity of F. sacchari. These results indicated that the pectate lyase gene reduced fungal pathogenicity by reducing the enzyme activity of the pathogen. Previous studies have shown that, in Colletotrichum coccodes, the reduced PL enzymatic activity levels in *CcpelA* gene-inhibited mutants were correlated with decreased pathogenicity (35). As expected, extracellular pectate lyase content was also significantly decreased in the *FsPL* knockout mutant compared to wild-type F. sacchari. These results indicated that the pectate lyase gene reduced fungal pathogenicity by reducing the enzyme activity of the pathogen. Thus, our results suggested that the *FsPL* gene increased F. sacchari virulence by enhancing the cell wall penetration and degradation abilities of this fungus.

The pectate lyase genes of several pathogens have been shown to induce necrosis when transiently expressed in plants using *Agrobacterium*-mediated transformation. For example, the transient expression of the pectate lyase genes from Phytophthora capsici (*PcPel*) and Verticillium dahlia (*VdPEL1*) caused cell death in pepper leaves and tobacco leaves, respectively (36, 37). Consistent with this, the *Agrobacterium*-mediated transient expression of *FsPL* caused cell death in tobacco leaves. As important components of secreted proteins, signal peptides may be required for protein recognition and the subsequent initiation of the PCD response in plants. For example, the signal peptides of Phytophthora capsici protein PcCBP3 and oomycete protein sPLD-like are required for the cell death induction activities of these proteins (38, 39). Previous studies in our research group have shown that the FsPL signaling peptide has secretory activity (23). To explore the effects of the signal peptide on the hypersensitive response and the induction of PCD, we compared necrosis degree between tobacco leaves transiently expressing *FsPL* and those transiently expressing the *FsPL* gene without the signal peptide. We found that the removal of the signal peptide reduced the size and severity of the necrotic lesions, indicating that the signal peptide was critical for the effective function of the *FsPL* gene.

The CWDEs secreted by some pathogens act both as virulence factors and inducers of plant immunity. For example, Phytophthora sojae GH12 protein *PsXEG1* is an important virulence factor but also acts as a PAMP to activate the plant PTI response (28). Similarly, the xylanase *BcXyl1* from Botrytis cinerea and the pectate lyase *VdPEL1* from Verticillium dahlia both increase fungal virulence and induce an immune response in plants (26, 37). As our results showed that *FsPL* was a virulence factor for F. sacchari and that *FsPL* induced cell necrosis in N. benthamiana leaves, we next aimed to determine whether *FsPL* also induced a PTI response in N. benthamiana. The transient expression of *FsPL* in tobacco leaves significantly upregulated several genes that are well-known markers of plant defense responses and significantly increased other markers of PTI, including ROS production, electrolyte leakage (as indicated by electric conductivity), and callose accumulation. A truncated *FsPL* (*FsPL*Δsp), in which the signal peptide was removed, was also expressed in N. benthamiana to determine whether signaling peptides were necessary for the induction of plant immune responses. The results showed that these PTI markers were less strongly increased after the transient expression of *FsPL* lacking the signal peptide. These results indicated that *FsPL* induced the PTI response in plants and that this response, to some extent, depended on the presence of the signal peptide. PCD and PTI responses induced by *FsPL* were attenuated by the loss of the signal peptide. Furthermore, we investigated the subcellular localization of GFP-tagged FsPL in N. benthamiana. The GFP signal was mainly observed at the cell edge (see Fig. S5 in the supplemental material). In a previous study, we demonstrated that the signal peptide of *FsPL* exhibited secretory function (23). Based on the finding that the removal of the signal peptide attenuated this response, it might be concluded that, in the agroinfiltrated leaves, *FsPL* is secreted in the apoplast.

The kinases BAK1 and SOBIR1 bind to most pattern recognition receptors, transmitting signals that activate downstream immune responses (40). For instance, the RLP23-SOBIR1-BAK1 complex mediates a MAMP-triggered immune response (19), as well as the cell death-inducing activity of *BcXyl1* (26, 41). *FsPL* did not induce necrosis in BAK1- or SOBIR1-silenced N. benthamiana, suggesting that these kinases are required for *FsPL*-mediated cell death and that *FsPL* induces the PCD response by activating the plant immune system.

In a previous study, the functions of genes in wheat leaves were verified using a particle bombardment method (41). Although we similarly attempted to verify the necrosis-inducing function of *FsPL* in sugarcane, the natural host of F. sacchari, we were unable to successfully express the necessary genes in the sugarcane leaves using particle bombardment. As an alternative, we used maize, a close relative of sugarcane. Overexpression of *FsPL* in maize leaves induced cell necrosis; *FsPL* without the signal peptide also induced cell necrosis in the maize leaves, but to a lesser degree. We therefore hypothesized that *FsPL* would likely also induce cell necrosis in sugarcane. In future studies, we intend to continue to attempt to overexpress *FsPL* in sugarcane to experimentally validate this hypothesis.

## MATERIALS AND METHODS

### Organisms, strains, and culture conditions.

Lab-cultured F. sacchari was used as the wild-type strain in this study. All F. sacchari strains, including the *FsPL* knockout mutants, were grown on potato dextrose agar (PDA; Solarbio, Beijing, China) or in potato dextrose water (PDW; Solarbio, Beijing, China) at 28°C. The Agrobacterium tumefaciens strain GV3101 (with pJIC_SARep), which was used for *Agrobacterium*-mediated transient gene expression in tobacco (Nicotiana benthamiana, N. benthamiana) leaves, was purchased from Shanghai Weidi Biotechnology Co., Ltd. (Shanghai, China). Escherichia coli strain TOP10 was used to propagate plasmids. Sugarcane cultivar Zhongzhe 1, which is sensitive to F. sacchari, was grown in a greenhouse at 28°C with a 16-h-light/8-h-dark photoperiod. Nicotiana benthamiana was grown in a growth chamber at 25°C also with a 16-light/8-h-dark photoperiod.

### Generation of the *FsPL-*deletion mutant (ΔFsPL).

First, a homologous recombinant fragment was constructed containing the sequences flanking the *FsPL* and the hygromycin gene (*Hyg*). The 5′- and 3′-flanking sequences of the *FsPL* gene, each approximately 1,000 bp long and designated A and B, respectively, were amplified from the genomic DNA of the F. sacchari wild-type strain using primers *FsPL*-AF/AR (see Table S1 in the supplemental material) and FsPL-BF/BR (Table S1). The two fragments were fused with the hygromycin gene using double-joint PCR (Fig. S2a), and the fusion fragments (about 4,200 bp) were transferred into F. sacchari protoplasts using polyethylene glycol (PEG)-mediated fungal genetic transformation as described previously (32). Hygromycin B (100 μg/mL) was used for screening.

### Southern blotting.

To verify that the F. sacchari *FsPL* deletion transformants were single-copy insertional mutations, genomic DNA was extracted from the deletion transformants and wild-type F. sacchari using the cetyltrimethylammonium bromide (CTAB) method. The genomic DNA samples from the wild-type F. sacchari and the transformants were digested with HindIII, and the *FsPL* probe (probe 1) was amplified from plasmid PVX-FsPL using primer pair *FsPL*-probe-F/*FsPL*-probe-R (Table S1). The *Hyg* probe (probe 2) was amplified from plasmid PUC19-*HYG* using primer pair *Hyg*-probe-F/*Hyg*-probe-R (Table S1). Southern hybridization experiments were then performed with a digoxin DNA labeling and detection kit (Roche DIG high prime DNA labeling and detection starter kit II), following the manufacturer’s instructions.

### Pathogenicity of the ΔFsPL F. sacchari mutants.

Ten sugarcane leaves, detached from 10 sugarcane plants, were scratched with a needle, inoculated with fungus plaque (diameter, 6 mm), and cocultured at 28°C. The necrosis of the leaves was observed and photographed. We next performed a cellophane penetration test (24) as follows. Fungal plaques of WT and ΔFsPL strains growing in PDA for 7 days were taken using a hole punch (diameter, 6 mm). The fungal plaques were inoculated onto cellophane-covered PDA plates. Three replicate plates were inoculated per strain. After incubation upside down in a constant temperature incubator for 3 days, the colonies were photographed. After removing the cellophane (including the mycelium) from the medium surfaces, the plates were replaced in the incubator for an additional 3 days to determine whether the mycelia had penetrated the cellophane. Plates were photographed at the end of the 3-day incubation.

### Pectate lyase assay.

The wild-type or mutant fungal plaques (diameter, 6 mm) were placed in conical flasks containing 100 mL PDW and incubated at 28°C with shaking at 220 rpm for 5 days. Three replicate experiments were performed in parallel. We used dinitrosalicylic acid (DNS) colometry (42, 43) to determine the pectate lyase content of the wild-type and mutant strains. To calculate d-galacturonic acid standard curves, solutions of different concentrations of d-galacturonic acid were prepared, and the absorbances of these solutions at 540 nm were assayed. Standard curves were constructed to show d-galacturonic acid content along the abscissa and absorbance at 540 nm along the ordinate, and the corresponding regression equation was calculated (Fig. S4). The extracellular pectate lyase activity levels in the ΔFsPL mutant and the wild type were obtained based on absorbance at 540 nm using the calculated regression equation.

### *Agrobacterium*-mediated transient expression of *FsPL* in N. benthamiana leaves.

Different fragments of FsPL (*FsPL*-FL, *FsPL*Δsp) were amplified using the cDNA of wild-type F. sacchari as the template. The fragments were inserted into ClaI/NotI-digested PVX using the in-fusion method and verified by sequencing (Sangon Biotech, Shanghai, China). The recombinant plasmids were transformed into A. tumefaciens strain GV3101 (pJIC SA_Rep), and the agroinfiltration assay was carried out in the leaves of N. benthamiana. The empty vector (PVX) and the recombinant empty vector (PVX-GFP) were used as negative controls. Because Bcl-2-associated X protein (BAX), a mouse proapoptotic protein, induces cell necrosis in N. benthamiana leaves that resembles the pathogen-induced hypersensitive response, leaf sites inoculated with PVX-BAX were used as positive controls. Inoculation sites were observed for 7 days and photographed on the seventh day after inoculation. ImageJ was used to analyze the lesion area (41). Two days after inoculation, the gene transcription and protein expression of the empty vector, GFP, BAX, *FsPL*, or *FsPL*-Δsp at each inoculation site were confirmed using RT-PCR and Western blot analysis. Each treatment was performed at least three times, using 12 leaves each time.

### RT-PCR, qRT-PCR, and Western blotting.

RT-PCR was used to detect the transcription of genes in plants. Total RNA was extracted from plant leaves 48 h after inoculation using TaKaRa MiniBest universal RNA extraction kits (TaKaRa, Beijing, China), following the manufacturer’s instructions. The total RNA was then used as a template for the synthesis of first-strand cDNA using a reverse transcription kit (Perfect Real Time; TaKaRa, Beijing, China). The cDNA template was used in subsequent PCRs to detect gene transcription.

The relative expression patterns of PCD- and SAR-related genes in plant leaves were detected using quantitative RT-PCRs (qRT-PCRs). The qRT-PCRs were performed using a LightCycler 96 system (Roche, Germany) and TB Green (TaKaRa Biomedical Technology, Beijing, China) in a fast two-step amplification. The internal reference genes used were NbEF-1 for N. benthamiana and actin for maize, and the relative expression levels of the target genes were calculated using the threshold cycle (2^−ΔΔ^*^CT^*) method (44).

Western blotting was used to detect the expression of the gene in N. benthamiana. Total proteins were extracted using radioimmunoprecipitation assay (RIPA) buffer (Solarbio, Beijing, China) and separated using SDS-PAGE. Gels were blotted onto polyvinylidene difluoride (PVDF) membranes (Millipore, Germany) with transfer buffer at 80 V for 3 h. Membranes were blocked for 2 h at room temperature and then washed. The anti-hemagglutinin (HA) antibody (1:1,000; Transgen, Beijing, China) was added, and the membranes were incubated at 4°C overnight. After incubation, the membranes were washed five times. Membranes were then incubated with goat anti-mouse antibody (1:1,000; Transgen, China) in the blotting buffer at room temperature for 2 h. Finally, the membranes were washed five times with Tris-buffered saline with Tween 20 (TBST) for 15 min each time. Signals were detected using Pierce ECL Western blotting substrate (Tiangen, China) in a ChemiDoc XRS+ system (Bio-Rad).

### Subcellular localization of FsPL in N. benthamiana.

The FsPL protein was predicted to be located in the cell wall and cell membrane at the subcellular level by Cell-PLoc 2.0 (Table S2). The FsPL protein was fused into the N terminus of the GFP protein, and the subcellular localization of the fusion protein was determined based on the transient expression of GFP in N. benthamiana (45).

### Induction of PTI in N. benthamiana by *FsPL*.

Three markers of PTI were measured in the inoculated N. benthamiana leaves, electrolyte leakage (as reflected by ionic conductivity), ROS production, and callose accumulation. To detect ionic conductivity, N. benthamiana leaf punches (diameter, 1 cm) were collected 48 h postinoculation. The leaf punches were placed in sterile deionized water and incubated at 25°C with shaking at 165 rpm for 2 h. After incubation, ionic conductivity was measured with a FiveEasy FP30 conductivity meter (Mettler-Toledo, Shanghai, China) (46). To detect ROS accumulation, N. benthamiana leaves were collected 48 h postinoculation, stained with 1 mg/mL 3′3-diaminobenzidine (DAB) (47), and decolorized with absolute ethanol to improve stain visibility. ROS accumulation was then observed using a microscope (Leica, Germany). To observe callose deposition, leaves were collected 48 h after inoculation, stained with 0.1% aniline blue, and examined under a fluorescence microscope (Zeiss, Germany) (48). Twelve leaves were used in each experiment. In the N. benthamiana leaves, the PVX and PVX-GFP inoculation sites were used as negative controls, while the PVX-BAX inoculation site was used as the positive control.

### Virus-induced gene silencing in N. benthamiana.

We used virus-induced gene silencing (VIGS) (49) to determine whether the immune-pathway genes *NbSOBIR1* and *NbBAK1* mediate *FsPL*-triggered cell death in N. benthamiana leaves. The target genes were inserted into EcoRI/BamHI double enzyme-digested pTRV2 vectors using the fusion method and verified by sequencing (performed by Sangon Biotech, Shanghai, China). The verified recombinant vectors were transformed into A. tumefaciens GV3101, and the strains carrying the pTRV2 constructs were mixed 1:1 with the strain carrying pTRV1 (49). The strain carrying pTRV2-GFP was used as a negative control; because the silencing of phytoene desaturase (PDS) in N. benthamiana leads to albinism, the strain carrying pTRV2-PDS was used as a positive control for the VIGS process. After 2 to 3 weeks of growth, the plants inoculated with pTRV2-PDS began to show obvious signs of albinism (Fig. S6), and leaves from the other inoculated plants were collected for RNA extraction and qRT-PCR. After confirming the successful silencing of each immune pathway gene in the N. benthamiana leaves using qRT-PCR, these leaves were infiltrated with the PVX-FsPL or PVX-BAX (positive-control) vectors. Phenotypic images were taken after 7 days of infection. Gene expression was detected using RT-PCRs and Western blots.

### Transient expression of *FsPL* in maize leaves using particle bombardment.

To further explore the functions of the *FsPL* gene, the single-barreled particle delivery system (41) was used to overexpress *FsPL* in the leaves of maize B73, which is closely related to F. sacchari host sugarcane. The *GUS* gene, which encodes β-glucosidase, was used as a reporter, as *GUS* hydrolyzes 5-bromo-4-chloro-3-indole-β-glucosidase (X-Gluc) into a blue substrate in living cells only (48). The open reading frames (ORFs) of *FsPL*, *FsPLΔsp*, *GUS*, and BAX were inserted into separate BamHI/KpnI doubly digested pCAMBIA2300 vectors. The plasmid mixtures were prepared into DNA packages (100 μL) for use. The apical meristem leaves of 1-month-old maize plants were cut into 6-cm-long segments, and the leaves were pasted on a 9-cm petri dish in parallel after the main veins were removed. To identify the optimal bombardment distance, we first prepared DNA packages (100 μL) containing the 2 μg GUS plasmid and then bombarded (8 μL/shot) the maize leaves with a PDS-1000/He (Bio-Rad) at distances of 6 cm, 9 cm, and 12 cm. As the greatest numbers of blue spots, corresponding to live cells, were observed in the leaves bombarded at 6 cm (Fig. S7), this distance was used in subsequent analyses. Maize leaves pasted into petri dishes were separately bombarded with the control and experimental plasmids (8 μL/shot) at a distance of 6 cm and a pressure of 1,100 lb/in^2^ using a PDS-1000/He (Bio-Rad). The bombarded leaves were dark cultured at 28°C for 2 days and then stained with 0.8 mg/mL X-Gluc (41) solution for 16 h. After decolorization in 100% ethanol, the blue dots on the leaves in each petri dish were counted. This experiment was replicated three times.

### Statistical analysis.

All experiments and data analyses were replicated at least three times. All data are presented as means ± standard deviations. All statistical analyses were performed using two-way analyses of variance (ANOVAs) in GraphPad Prism 8.2.1 (GraphPad Software, USA).
