# Supplementary material for: Pectate Lyase from Fusarium sacchari Induces Plant Immune Responses and Contributes to Virulence
Source: Microbiol Spectr. 2023 May 4;11(3):e00165-23. doi: 10.1128/spectrum.00165-23 (PMC10269888; doi:10.1128/spectrum.00165-23)
Supplement: Supplemental file 1 — Fig. S1 to S7 and Tables S1 and S2. Download spectrum.00165-23-s0001.docx, DOCX file, 1.6 MB [file spectrum.00165-23-s0001.docx]

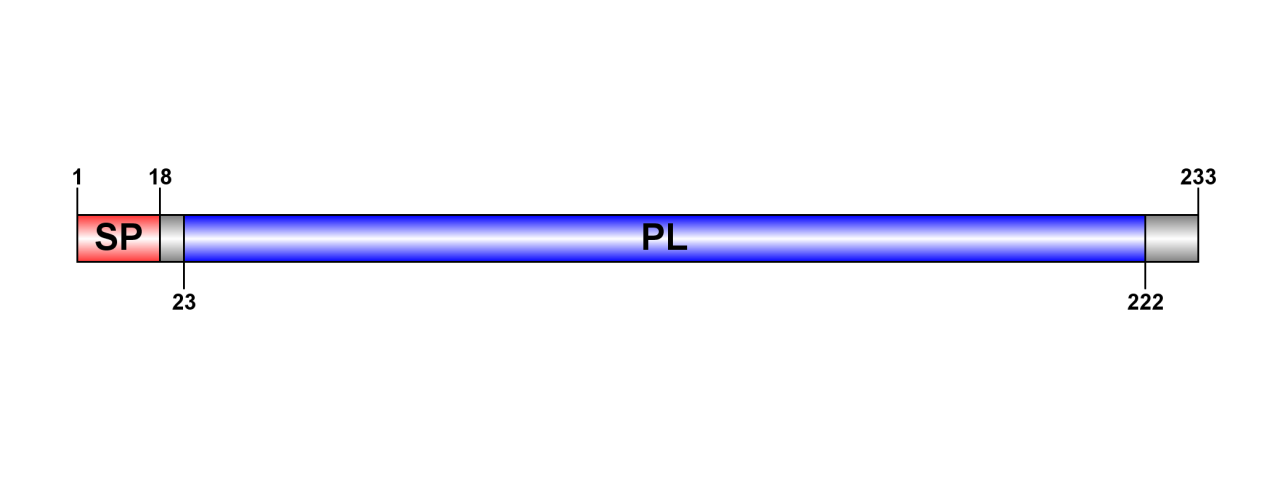


**Figure S1** Amino acids sequences of FsPL. IBS 1.0 was used to construct the schematic diagram. SP: signal peptide. PL: the conserved pectate lyase domain.


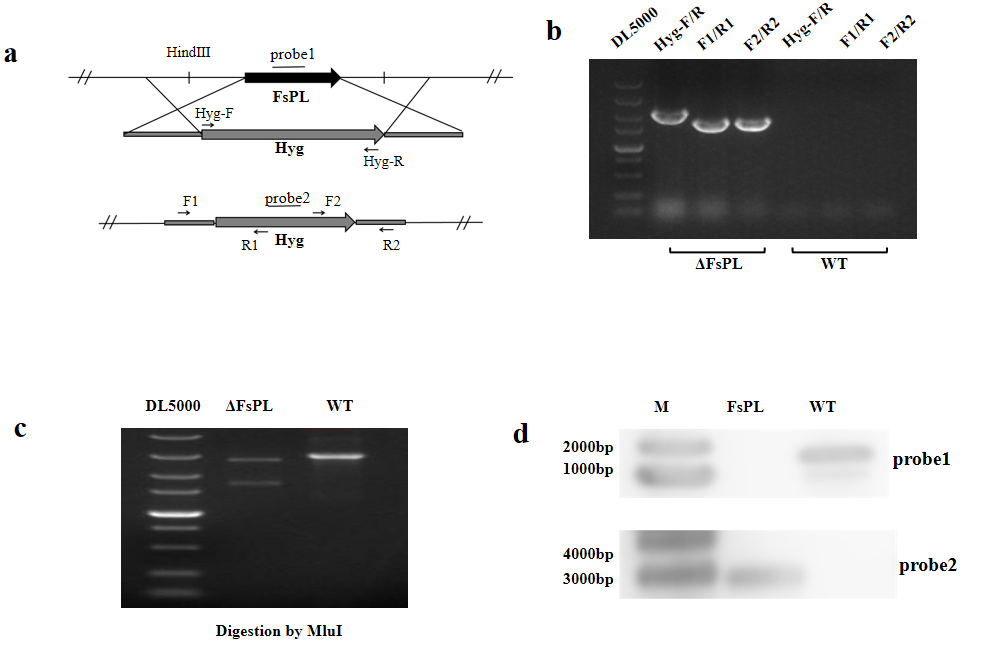


**Figure S2** Acquisition and validation of the ΔFsPL mutant. (a) Schematic showing the deletion of the *FsPL* gene in *Fusarium sacchari*. (b) Identification of the successful deletion mutants using agarose gel electrophoresis of the PCR products. Lanes: DL5000, DNA ladder; *Hyg*-F/R, primers *Hyg*-F/*Hyg*-R for the hygromycin resistance gene; F1/R1, F1 was derived from the sequence upstream of the *FsPL* gene and R1 was derived from the *Hyg* gene sequence; F2/R2, F2 was derived from the *Hyg* gene sequence and R2 was derived from the sequence downstream of the *FsPL* gene. (c) Digestion using MluI. The MluI digestion site, which exists in the *Hyg* gene sequence but does not exist in the *FsPL* gene sequence, was selected. The long sequences of the wild type and the transformants (A-FsPL-B and A-*HYG*-B) were amplified using the *FsPL*-AF and *FsPL*-BR primers and verified by digestion with the enzyme MluI. (d) Southern blots showing the genomic DNA of the wild-type and ΔFsPL mutant after digestion with Hind III and hybridization with probe 1 and probe 2.


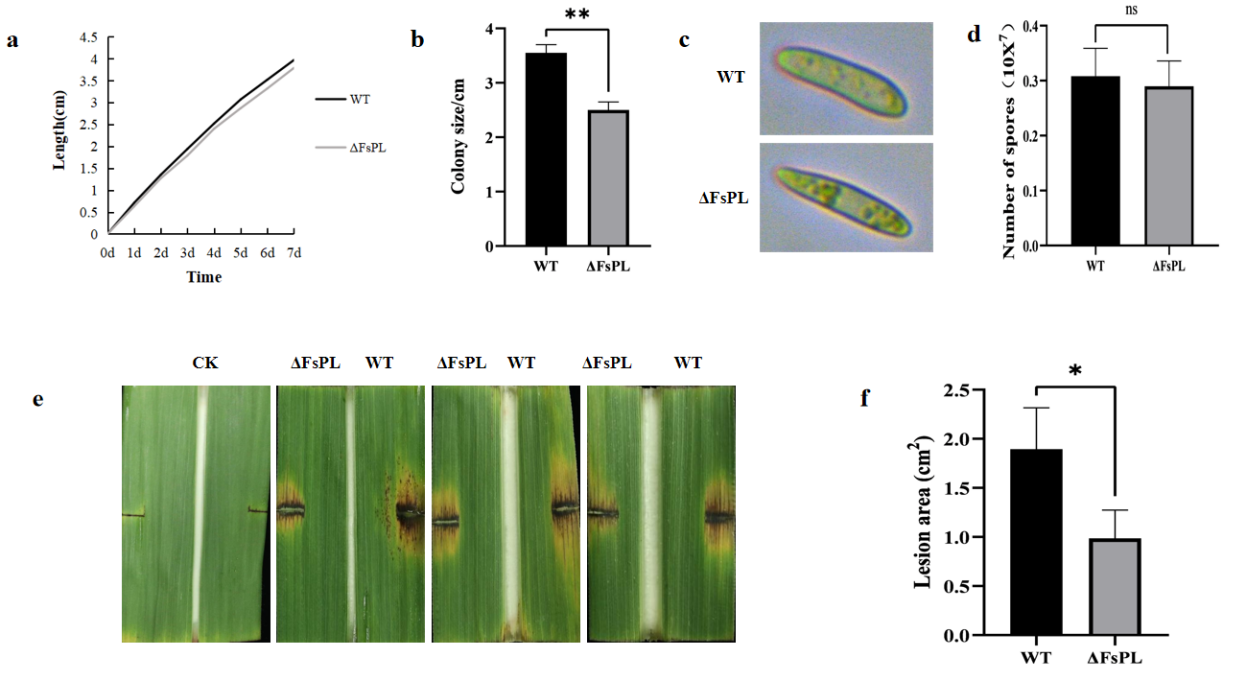


**Figure S3** Phenotype and pathogenicity of the ΔFsPL mutant. (a) Mycelial growth curves for wild-type *Fusarium sacchari* and the ΔFsPL mutant. To determine the growth rates of the wild type and the ΔFsPL mutant, we transferred the WT and ΔFsPL fungal plaques, each 6 mm in diameter, to plates containing 15 mL PDA. Each culture was incubated inverted at 28°C in the dark for 7 d. Colony diameters were measured every 24 h, and mycelial growth curves were drawn based on the recorded data. (b) Colony size (radius /cm) of the wild type (WT) and mutant isolate (ΔFsPL) on PAM. (c) Morphology of wild-type (WT) and ΔFsPL spores. (d) Spore production by the wild type and the ΔFsPL. The fungal plaque (6 mm) of each strain to be tested was transferred to carboxymethylcellulose sodium (CMC) medium, and conidial suspensions were obtained after shock culture at 28°C for 3 d. After 3 d, the number of conidia were counted using a blood-cell counting plate. (e) Representative images of detached sugarcane leaves (cultivar “Zhongzhe 1”) 3 d after inoculation with wild-type *F. sacchari* (WT), the ΔFsPL mutant, or double-deionized water as a mock control (CK). (f) Quantification of the results shown in panel d.


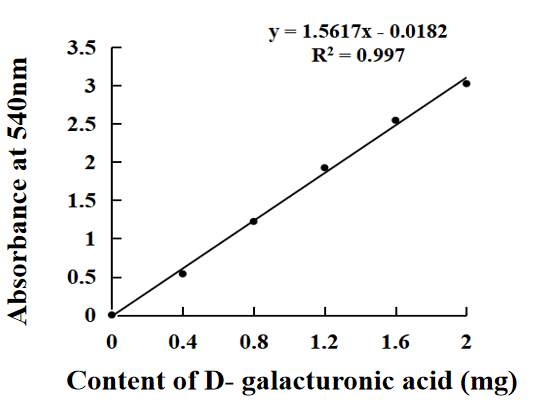


**Figure S4** Standard curve showing absorbance at 540 nm as a function of D-galacturonic acid content. The corresponding regression equation was y = 1.5617x-0.0182 (R^2^ = 0.997).


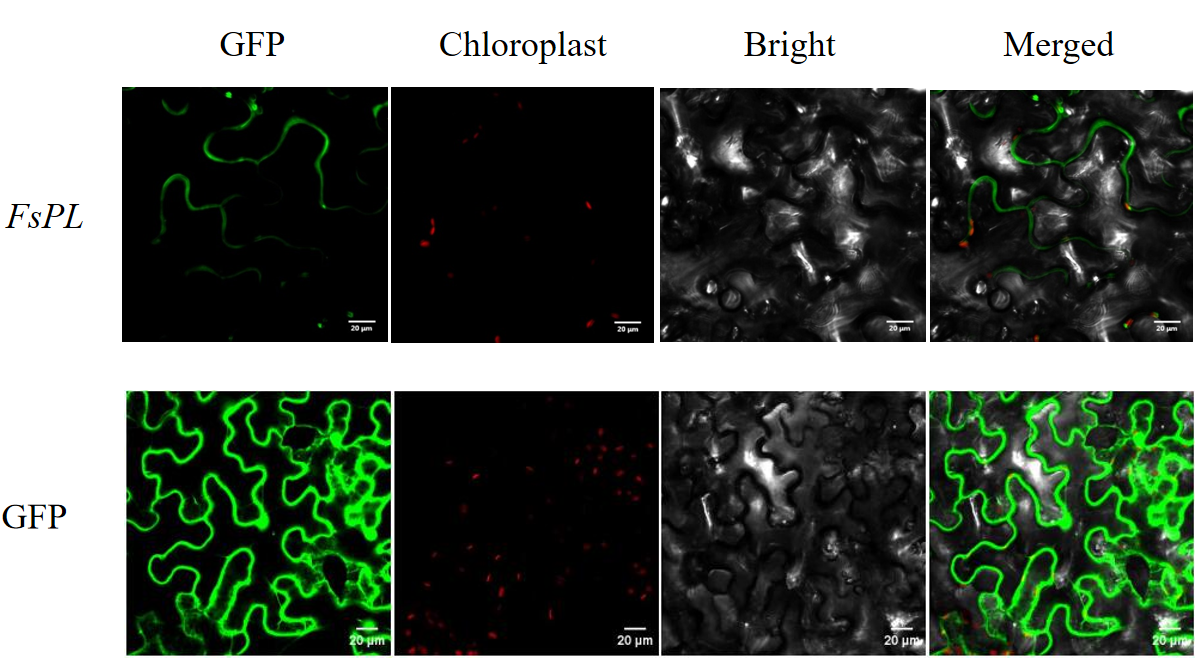


**Figure S5** Subcellular localization FsPL proteins in *Nicotiana benthamiana*. The N-terminal of the FsPL-GFP fusion protein was cloned into the pBWA(V)HS expression vector and transiently expressed in *N. benthamiana*. The subcellular localization of each protein was determined with a confocal laser-scanning microscope based on GFP (488 nm excitation and 512 nm emission wavelengths), chloroplast auto-fluorescence (using 488 nm excitation and 656 nm emission wavelengths), bright field, and merged.


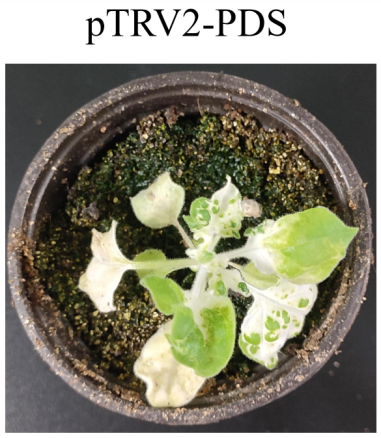


**Figure S6** *N. benthamiana* albinism appears after PDS gene silencing. Representative example of *PDS-*silenced *N. benthamiana*, showing the albinism exhibited after 2–3 weeks of growth.


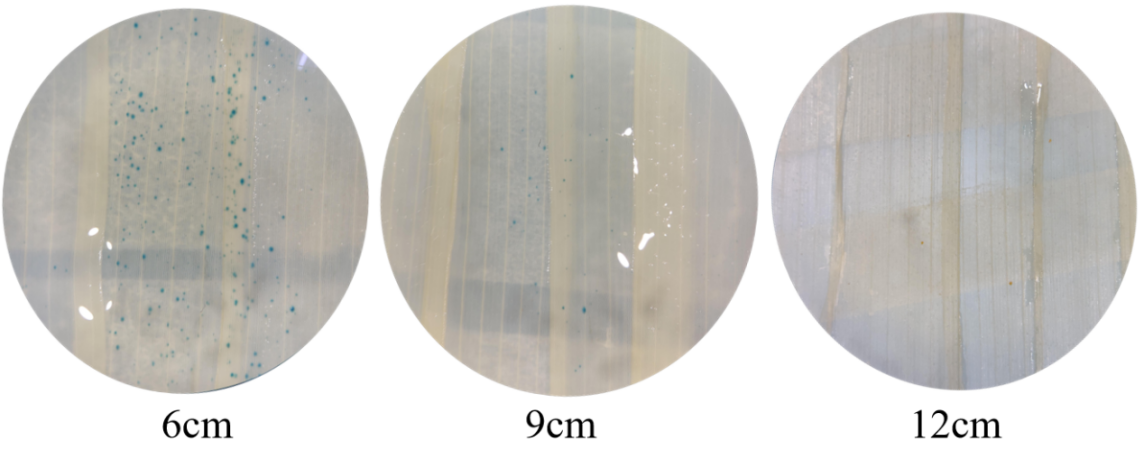


**Figure S7** Identification of optimal bombardment distance. Representative images showing maize leaves after bombardment with the positive control plasmid at distances of 6, 9, and 12 cm.

**TABLE S1** Primer sequences used in this study

| **Primer name** | **Sequence (5'-3')** | **Purpose** |
| --- | --- | --- |
| FsPL-AF | GACTGGCGAAATAACCAGATCGA | Amplification of the upstream fragment of *FsPL* |
| FsPL-AR | tctttctagaggatccccgggtacATGTTTGTATCGGCTTGATATTGCTC |  |
| FsPL-BF | atatcatcttctgtcgacctgcaggTCTGGCGGTTGGGAGAGT | Amplification of the downstream fragment of *FsPL* |
| FsPL-BR | CGAGGCATTCTTGGGTCATTTTAG |  |
| *Hyg*-F | CGGTACCCGGGGATCCTCTAG | Amplification of the full-length *Hyg* gene |
| *Hyg*-R | GCCTGCAGGTCGACAGAAGATG |  |
| FsPL-F1 | GGAGCTTGGCGTTACCTGTC | Translocator detection primer |
| *Hyg*-R1 | GGGAGACGAGATCAAGCAGAT |  |
| *Hyg*-F2 | TCGCGCATATGAAATCACGC | Translocator detection primer |
| FsPL-R2 | CCTCCCGCTCCAATAAGCAT |  |
| FsPL-probe-F | CTTCGACGCAAAGTGGGTTC | Amplification of the *FsPL* probe (probe1) |
| FsPL-probe-R | CTCCATGTGGACTGATCGCT |  |
| *Hyg*-probe-F | TCGGTTTCAGGCAGGTCTTG | Amplification of the *Hyg* probe (probe2) |
| *Hyg*-probe-R | CTCGGAGGGCGAAGAATCTC |  |
| FsPL-ClaI-F | agcaccagctagcatcgatATGCACGCCTCCAGCCT | Clone *FsPL* to PVX for expression in *N. benthamiana* |
| FsPL-NotI-R | atcgtatgggtacgcggccgcCTAGCACTTGCCAGCCTTGG |  |
| FsPLΔsp-ClaI-F | agcaccagctagcatcgatATGTGTCTCGGCTACACCGGC | Clone *FsPL*Δsp to PVX for expression in *N. benthamiana* |
| FsPLΔsp-NotI-R | atcgtatgggtacgcggccgcCTAGCACTTGCCAGCCTTGG |  |
| GFP-ClaI-F | agcaccagctagcatcgatATGAGTAAAGGAGAAGAACTTTTCACTGG | Clone GFP to PVX for expression in *N. benthamiana* |
| GFP-NotI-R | atcgtatgggtacgcggccgcTTTGTATAGTTCATCCATGCCATGTGTAAT |  |
| BAX-ClaI-F | agcaccagctagcatcgatATGGACGGGTCCGGGGA | Clone BAX to PVX for expression in *N. benthamiana* |
| BAX-NotI-R | atcgtatgggtacgcggccgcGCCCATCTTCTTCCAGATGGTG |  |
| NbEF1*-*qPCR-F | TGGTGTCCTCAAGCCTGGTAT | qPCR |
| NbEF1-qPCR-R | ACGCTTGAGATCCTTAACCGC |  |
| NbHIN1-qPCR-F | CCAACTTGAACGGAGCCTATTA |  |
| NbHIN1-qPCR-R | AGGCATCCAAAGAGACAACTAC |  |
| NbHSR203J-qPCR-F | ACGCAGATTTCAACCGAGTAT |  |
| NbHSR203J-qPCR-R | GCCAGTCGCATTGGAGATAA |  |
| NbPR2-qPCR-F | AGGTGTTTGCTATGGAATGC |  |
| NbPR2-qPCR-R | TCTGTACCCACCATCTTGC |  |
| NbLOX-qPCR-F | AAAACCTATGCCTCAAGAAC |  |
| NbLOX-qPCR-R | ACTGCTGCATAGGCTTTGG |  |
| NbERF1-qPCR-F | GCTCTTAACGTCGGATGGTC |  |
| NbERF1-qPCR-R | AGCCAAACCCTAGCTCCATT |  |
| GFP-EcoRI-F | tgagtaaggttaccgaattcATGAGTAAAGGAGAAGAACTTTTCACTGG | VIGS in *N. benthamiana* |
| GFP-BamHI-R | gtgagctcggtaccggatccTTTGTATAGTTCATCCATGCCATGTGTAAT |  |
| BAK1-EcoRI-F | tgagtaaggttaccgaattcCTAACAGGTCAACTGGTACCACAGC |  |
| BAK1-BamHI-R | gtgagctcggtaccggatccTGGTCCTGTCAAATGATTGCTTGAGAG |  |
| SOBIR1-EcoRI-F | tgagtaaggttaccgaattc CAACCCCCAATGGATGCTGCA |  |
| SOBIR1-BamHI-R | gtgagctcggtaccggatccAGTAGCTATCCCTACTGCAATTCGG |  |
| PDS-EcoRI-F | tgagtaaggttaccgaattcGAACATATTGAGTCAAAAGGTGGCC |  |
| PDS-BamHI-R | gtgagctcggtaccggatccGCTTCTGCTGAAGAGCAGATTATC |  |
| BAK1-qPCR-F | CGCTTCCTGAGGCTGAATAATA | qPCR |
| BAK1-qPCR-R | GGTCCTGTCAAATGATTGCTTG |  |
| SOBIR1-qPCR-F | GGCCAGACTGCCATTACTT |  |
| SOBIR1-qPCR-R | GTGTCGTCCCAACCAATCTAA |  |
| FsPL-BamHI-F | aggtcgactctagaggatccATGCACGCCTCCAGCCT | Clone *FsPL* to pCAMBIA2300 for expression in maize |
| FsPL-Kpn I-R | acatacgcgtggtaccCTAGCACTTGCCAGCCTTGG |  |
| FsPLΔsp-BamHI-F | aggtcgactctagaggatccATGTGTCTCGGCTACACCGGC | Clone *FsPLΔsp* to pCAMBIA2300 for expression in maize |
| FsPLΔsp-KpnI-R | acatacgcgtggtaccCTAGCACTTGCCAGCCTTGG |  |
| BAX-BamHI-F | aggtcgactctagaggatccATGGACGGGTCCGGGGA | Clone BAX to pCAMBIA2300 for expression in maize |
| BAX-KpnI-R | acatacgcgtggtaccGCCCATCTTCTTCCAGATGGTG |  |
| Actin-qPCR-F | GGTTTCGCTGGTGATGATGC | qRT-PCR |
| Actin-qPCR-R | CAATGCCATGCTCAATCGGG |  |
| ZmLOL2-qPCR-F | CGTCGTCGTCAGCGATTAAC |  |
| ZmLOL2-qPCR-R | CGGGCAGGCTAGCGTATTTA |  |
| ZmPR1-qPCR-F | CCTACGGCGAGAACCTCTT |  |
| ZmPR1-qPCR-R | TCGTAGTACTGCTTCTCGGACA |  |
| ZmPR5-qPCR-F | ATCGGCCGGAATAGGCTCTG |  |
| ZmPR5-qPCR-R | CGCGTACATACAAATGCGTGC |  |

**TABLE S2** [Subcellular](D:/LenovoSoftstore/Install/wangyiyoudaocidian/8.9.6.0/resultui/html/index.html#/javascript:;) [localization](D:/LenovoSoftstore/Install/wangyiyoudaocidian/8.9.6.0/resultui/html/index.html#/javascript:;) of FsPL vector

| Gene | [Subcellular](D:/LenovoSoftstore/Install/wangyiyoudaocidian/8.9.6.0/resultui/html/index.html#/javascript:;) [localization](D:/LenovoSoftstore/Install/wangyiyoudaocidian/8.9.6.0/resultui/html/index.html#/javascript:;) |
| --- | --- |
| *FsPL* | Cell membrane and cell wall |
